# Pathological Fractures in Aneurysmal Bone Cysts: A Systematic Review

**DOI:** 10.3390/jcm13092485

**Published:** 2024-04-24

**Authors:** Doriana Di Costa, Elena Gabrielli, Mariagrazia Cerrone, Emidio Di Gialleonardo, Giulio Maccauro, Raffaele Vitiello

**Affiliations:** 1 Department of Orthopaedics, Fondazione Policlinico Universitario Agostino Gemelli IRCCS, 00168 Rome, Italy; doriana.dicosta01@icatt.it (D.D.C.); mariagrazia.cerrone01@icatt.it (M.C.); emidio.digialleonardo01@icatt.it (E.D.G.); giulio.maccauro@policlinicogemelli.it (G.M.); raffaele.vitiello@guest.policlinicogemelli.it (R.V.); 2 Department of Orthopaedic Surgery, Università Cattolica del Sacro Cuore, 00168 Rome, Italy

**Keywords:** aneurysmal bone cyst, pathological fracture, benign tumors

## Abstract

**Background:** Aneurysmal bone cysts (ABCs) are osteolytic, non-malignant, vascular lesions of the bone. Pathological fractures can be a manifestation of the ABCs, which occur in about 8% of ABCs. Different treatments have been described in the literature, but, nowadays, an optimal management of the pathological fractures in patients with ABCs is still a matter of debate and there are no standard guidelines for treatment nor any shared indication about the best surgical intervention. The aim of our study is to review the current literature available on this matter exploring and confronting different surgical treatments for pathological fractures in ABC in order to clarify the surgical approach to these patients. **Methods:** A systematic review of the literature indexed in PubMed, MEDLINE, and Cochrane Library databases was carried out. The Preferred Reporting Items for Systematically Reviews and Meta-Analyses (PRISMA) were followed. **Results:** A total of 37 articles were relevant and were finally included in the study. In total, we reached a population of 140 patients. Of the 140 patients included in the review, 124 patients (88.6%) underwent curettage surgery, 15 patients (10.7%) underwent en bloc resection surgery. A total of 47% of patients (70) underwent synthesis surgery with a plate, screw, nail, or external fixator. Adjuvant treatments were used in 8.6% of patients (12). Complications involved 20.7% of the patients (29). **Conclusions:** In conclusion, the treatment of pathological fractures in aneurysmal bone cysts requires careful patient assessment, considering factors such as age, the presence of open growth plates, the location of the lesion, and the surgeon’s expertise.

## 1. Introduction

Aneurysmal bone cysts (ABCs) are rare, non-malignant osseous, tumor-like, vascular lesions [1]. ABCs were described for the first time in 1942 by Jaffe and Liechtenstein [2]. The majority of patients diagnosed with an aneurysmal bone cyst are children and adolescents. The mean age of presentation is approximately 18 years old, with a preponderance in the female sex [3].

ABCs represent 9.1% of all bone tumors [4]. The most frequent localizations are lower limbs (50–60%), with tibia representing almost 40% of the cases [5], the spine is involved in almost 15% of the cases and the pelvis in 9% [1]. Typically, ABCs are located near the metaphysis, adjacent to the physis region, and eccentrically [6]. This last characteristic can be useful to make a differential diagnosis of ABC or unicameral bone cyst radiographically [7].

The etiology of aneurysmal bone cysts is uncertain; they seem to be linked to vascular malformations in the bone, but this consideration alone is not enough to fully understand their development. There are different theories about ABCs etiology. The first hypothesis is that they form as a primary tumor, the second explanation is that they develop in the site of a previous trauma [8], while the third theory suggests to consider ABCs as a secondary lesion to another primary bone tumor, such as a giant cell tumor or chondroblastoma [9].

The histopathology of ABCs is characteristic: they are composed of a blood-filled cavity within an expanded region of the bone [10]. The shell of the cysts is made by reactive bone, without endothelium. The absence of endothelium makes the name “cyst” improper. Upon histopathological examination, mitotic figures are commonly seen in the lesions, but atypical figures should not be present [11]. Today, a biopsy is considered mandatory to confirm the diagnosis of ABCs [12].

In the diagnostic iter, the first exam that is usually proposed to the patient is an X-ray, that usually shows a well-defined osteolytic lesion with a soap-bubble appearance. The second level of investigation is magnetic resonance, which shows cystic formations with typical fluid–fluid levels due to blood sedimentation [12].

ABC behavior is unclear. They can evolve in three different ways: they can be locally aggressive and, in these cases, they can even be mistaken for malignant tumors, but ABCs can also grow up slowly or even be quiescent. In some cases, ABCs have shown a tendency towards spontaneous resolution [4].

Clinically, ABCs can present with pain as the first symptom at the onset, with a possible palpable mass or even deformity [13]. Pathological fractures can also be a manifestation of the ABC both at the onset, as the first presentation of the tumor, or during the follow-up of a known lesion. Local erosion of the bone tissue can be documented when ABCs grow up aggressively, leading to pathological fractures. Because of ABCs metaepiphyseal localization, in children, they could result in a growth-plate injury, which could lead to misalignment. Studies show that even patients without evident growth-plate involvement have a 10% risk of a subsequent disturbance of growth [14].

Pathologic fracture occurs in about 8% of ABCs, but the incidence may be as high as 21% in ABCs that have spinal involvement [5]. A pathological fracture is caused by minor trauma that typically cannot cause a bone fracture in healthy bone. It develops in bone that has been damaged and debilitated via a pathological process, with the result of loss of normal strength and structural integrity [15].

We can find different treatments for ABCs described in the medical literature. The primary form of treatment used is intralesional curettage, with or without adjuvants. A bone graft or bone cement, like polymethylmethacrylate (PMMA), is commonly used after curettage to reconstruct the bone’s defect [7,16]. Adjuvant agents can be used for percutaneous intralesional injection, which represents another important therapeutic possibility. It appears that adjuvants can improve the local control rate when combined to curettage [9].

The most used adjuvants, for both intralesional curettage and percutaneous intralesional injection, are sclerosant agents, for example, Polidocanol and Ethibloc. Sclerotherapy has been proven to be a safe method, associated with good local control and few side-effects [17,18]. Another possible adjuvant agent is doxycycline, due to its antineoplastic properties [19]. It is also possible to use the systemic application of denosumab or bisphosphonates, but only a preliminary study was found in this field [20]. Alternatively, when surgical resection is difficult, embolization can be used as a adjuvant or single therapy but is often technically demanding.

Patients with a pathological fracture require surgery to treat the ABC and to stabilize the fracture. The first step is curettage, or wide resection, of the lesion and performing the graft: PMMA, autograft, and allografts can be used to fill the cavity, similarly used in the ABCs’ treatment without fracture. The use of internal fixation depends on the fracture’s location and displacement [21].

Different treatments have been described in the literature, but, nowadays, optimal management of the pathological fractures in patients with ABCs is still a matter of debate and there are no standard guidelines for treatment nor any shared indication about the best surgical intervention.

The aim of our study is to review the current literature available on ABC fracture treatment and confront different surgical treatments in order to clarify the surgical approach to these patients.

## 2. Materials and Methods

A systematic review of the literature indexed in PubMed, MEDLINE, and Cochrane Library databases using the search terms “Fracture” and “Aneurysmal Bone Cyst” was carried out. To minimize the number of missed studies, no filters were applied to the search strategy. The bibliography of the selected studies was accurately searched by hand, to identify further studies not found during the electronic search. No restrictions were applied concerning the date of publication nor the language. The title of the journal, name of authors, or supporting institutions were not masked at any stage.

**Figure 1:**
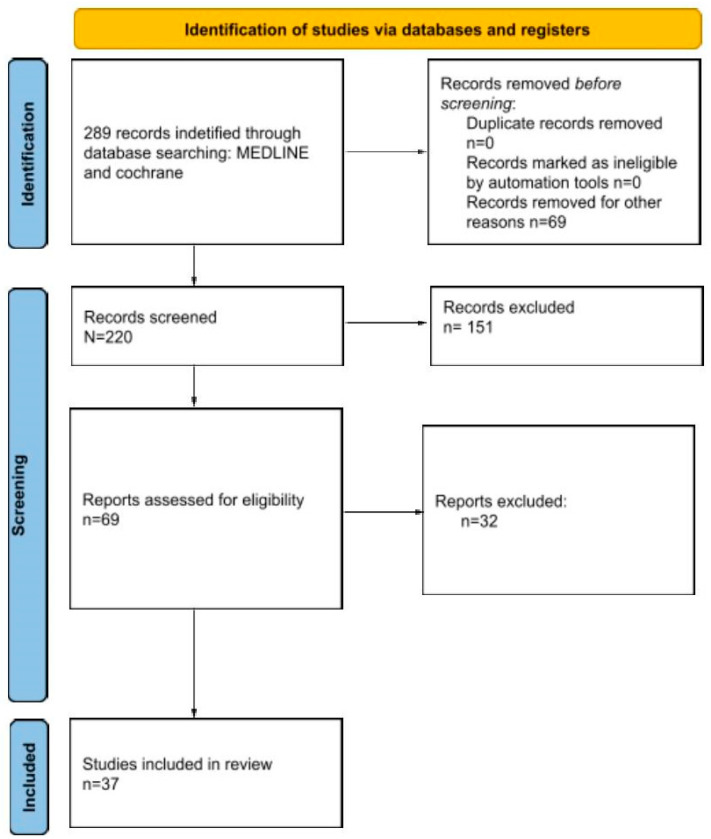
PRISMA 2020 flow diagram for new systematic reviews, which included searches of databases and registers only.

The Preferred Reporting Items for Systematically Reviews and Meta-Analyses (PRISMA) were followed, as reported in Figure 1. In order to be considered for this review, the articles needed to present some inclusion criteria: at least a section of the population understudy needed to be affected by an aneurysmal bone cyst, bone fracture, and the treatment performed needed to be explicit.

Abstracts and full texts were independently screened by two authors (E.G and E.DG), any discordance was solved via consensus with a third author (D.DC). Case reports were included in the review, while systematic reviews and meta-analyses were excluded. The methodological quality of the studies was assessed using the modified Coleman methodology score (mCMS). Each article was evaluated by two independent investigators (M.C and D.DC); in cases with more than a five-point difference between their rating, the discrepancy was solved via consensus with a third author (R.V). The mCMS ranged from 0 to 100 points, representing a well-designed study with no bias or confounding factors. All the selected studies were retrospectively analyzed by an author (E.DG) who then extracted and entered the data in an Excel worksheet. The collected data included the following: main author, year of publication, mCMS, number of patients, patient age and gender, aneurysmal cyst site, type of surgery, complications, healing rate, and follow-up. Lastly, the data sheet was reviewed by two authors (G.M and R.V) who agreed on the extracted data.

## 3. Results

### 3.1. Demographic Data

A total of 289 articles resulted from the search. Following the Prisma flow-chart, 37 articles were relevant and were finally included in the study. According to the mCMS evaluation, the mean score of the studies reached was 36 points (10–87 points).

In total, we reached a population of 140 patients. In 6 studies (*n* = 34), the gender of the patients was not specified. From the extrapolated data on the sex of the patients, 64 were male and 42 females. The average age among the various studies considered was 18.70 years (ranging from 2 years to 69 years) [Table 1].

The mean radiological and clinical follow-up of the patients considered was 44.06 months (ranging from 1 month to 330 months). In only two studies, by Jackson et al. [22] and Schmitz et al. [23], the duration of follow-up was not reported.

No clinical or functional scores were considered because only 8/37 articles reported one.

**Table 1:** Demographic data.

Authors	Year of Publication	Number of Patients	Mean Age (Years)	Modified Coleman Score
Sonkusale et al. [24]	2023	1	13	37
Pai et al. [25]	2022	1	5	22
Teixeira-Vaz et al. [26]	2022	1	7	39
Kushwaha et al. [27]	2021	1	13	24
Tomaszewski et al. [28]	2020	6	10.8	65
Weber et al. [29]	2019	1	22	27
Trăilescu et al. [30]	2019	3	11.5	28
Dorosh et al. [31]	2019	1	4	29
Chhawra et al. [32]	2019	1	40	34
Okuda et al. [33]	2019	1	15	40
Kirker et al. [34]	2019	1	4	71
Purohit et al. [35]	2019	1	15	38
Rahman et al. [36]	2018	4	12	87
Panchwagh et al. [37]	2018	4	26.5	75
Arif et al. [38]	2016	1	25	28
Ferreira et al. [39]	2016	1	2	15
Plaiker et al. [40]	2016	1	23	12
Erol et al. [41]	2015	64	10	65
Welk et al. [42]	2014	1	12	15
Geffroy et al. [43]	2012	2	13	36
Babazadeh et al. [44]	2011	1	21	29
Rapp et al. [45]	2011	2	14,75	39
Rossi et al. [46]	2010	1	5	32
Grzegorzewski et al. [47]	2010	2	22	39
Xu et al. [48]	2009	1	20	38
Nydick et al. [49]	2009	1	11	15
Beris et al. [50]	2009	1	21	53
Clayer et al. [51]	2008	15	9.5	57
Lampasi et al. [52]	2007	1	7	53
Goddard et al. [53]	2007	1	40	20
Jackson et al. [22]	2007	3	12.2	10
Schmitz et al. [23]	2005	1	37	25
Ortiz et al. [54]	2005	9	9	27
Session et al. [55]	2005	1	20	35
Gailloud et al. [56]	2002	1	58	29
Snell et al. [57]	2001	1	10	29
Yamamoto et al. [58]	2000	1	69	20

### 3.2. Aneurysmatic Bone Cyst Location and Treatment

In 73 patients (52%), the site of the aneurysmal bone cyst was in the lower limbs. The femur is the most-affected bone segment in the lower limbs with 58 cases of ABC fracture (79.5%). In forty-seven patients, the proximal femur is involved: in six patients the cyst affected the distal femur, in two patients the femoral dialysis was affected, and in three patients the femoral site was not specified.

In 40 patients (28.6%), the location of the cyst was at the level of the upper limb. The most frequent localization to the upper limb is the humerus with 33 cases (82.6%). In seven patients (4.5%), the site of onset was at the level of the spine, with the cervical segment being affected in 57% of cases.

Other sites were the mandible—one case (0.7%) ileum—five cases (3.3%), ischium—two cases (1.30%), and pubis—three cases (2%). In the study by Ortiz et al. including nine patients, the site of the cyst was not specified.

Of the 140 patients included in the review, 124 patients (88.6%) underwent curettage surgery and 15 patients (10.7%) underwent en bloc resection surgery. Only one patient (1%) in the study of Rossi et al. [46] underwent arterial embolization surgery, N-2-butyl-cyanoacrylate was used as an embolizing agent. A total of 47% of patients (70/140) underwent synthesis surgery with a plate, screw, nail, or external fixator. The percentage of patients underwent plate synthesis is 21.4% (15/70). There were eight patients who underwent nail synthesis, representing 10% (8/70) of the patients who underwent synthesis. Only one patient, described in the study by Kushwaha et al. [27], was treated using an external fixator, with the duration of treatment being 12 weeks. Only one patient, described in the study by Xu et al. [48], was treated using screws. Of the patients with vertebral localization, five underwent arthrodesis surgery. In 40 patients, it was not possible to extrapolate which type of synthesis was used.

In 78 patients, the lesion was filled with autologous/allogeneic bone. In nine patients, a combination of synthetic bone and autologous or allogenic bone was used. In 24 patients, synthetic bone was used; meanwhile, in thirteen patients, the type of graft used was not specified. In four patients, cement was used as a lesion filler.

Adjuvant treatments were used in 8.6% of patients (12/140). Liquid nitrogen injections were used in four patients. Phenols were used in two patients. In one patient, a combination of phenol and alcohol was used as an adjuvant treatment. In two patients, autologous platelet-rich plasma was used. One patient underwent electrocauterization. In one patient, a combination of calcitonin and methylprednisolone was used. One patient was treated with cryotherapy [Table 2 and Table 3].

In 12 studies, comprising 23 patients, the period during which patients observed a weight-bearing ban was reported; the mean time was 11 weeks (ranging from 3 weeks to 56 weeks).

### 3.3. Complications and Healing Rate

Fourteen authors reported complications in their studies. Complications involved 20.7% of the patients (29/140): recurrences in 5.7% (8/140), shortening of the affected bone segment in 3.6% (5/140), non-synthesis fractures in 2.1% (3/140), angular deformities in 3.6% (5/140), residual pain in 1.4% (2/140), no healing/partial healing in 2.9% (4/140), neurological deficit 0.7% (1/140), and ROM limitation in 0.7% (1/140).

The most frequently encountered complication was residual recurrence (8/28). Only two studies, by Clayer et al. [51] and Ortiz et al. [54], reported a cure rate lower than 100%, 90% and 88%, respectively. In two studies, by Goddard et al. [53] and Jackson et al. [22], it was not possible to extrapolate the healing rate. The average healing rate between the various studies was 99.37%.

In nine studies with a total of thirty patients, healing times were reported with an average healing time of 7.88 months; however, at the end of follow-up there was 100% healing, except in two studies.

Of the patients who presented with recurrence of ABC (eight patients), seven underwent retreatment with resolution of the picture, while in one patient there was spontaneous resolution.

Of the patients who reported fracture as a complication (*n* = 3), it was not specified whether they were reoperated upon.

Patients with angular deformity (*n* = 5) underwent reoperation in only one case.

In the patients with partial healing or no healing (*n* = 4), only one was reoperated upon, while conservative treatment was chosen in another case. In two cases, it was not specified if they were operated upon or not [Table 4].

## 4. Discussion

The ABC is an osteolytic lesion of the bone characterized by blood-filled cavities separated by connective tissue septa containing fibroblasts, mononuclear cells, osteoclast-type giant cells, and reactive woven bone [59]. In more than half of the cases, they are localized in long bones, such as the tibia and femur, particularly at the metaphyseal level; 12-30% cases affect the spine; and, in the remaining cases, flat bones such as the pelvis are involved [60,61]. In our analysis, we reported similar percentages, with the exception of spine localization being less frequent compared to the literature. They typically manifest with pain and swelling, while, to a lesser extent, they may be associated with pathological fractures in about 8% of cases [5].

As of today, in the literature, there is still an open debate regarding the most appropriate treatment for ABCs, particularly when they are associated with pathological fractures and many different strategies are applied, as the data that we collected show, in the absence of a guideline to follow. Curettage is considered the standard treatment for ABCs and it is, in fact, the most used strategy among all patients considered in our review [28,29,60,62]. Some authors reserve en bloc excision only for recurrent cases or for lesions that can be removed surgically without bone reconstruction [7,63]. These two different surgical approaches lead to different complications, particularly according to the literature on en block excision resulting in a lower recurrence rate: none of the considered patients who underwent this kind of surgery presented recurrence at the follow-up compared to the ones treated with curettage who presented recurrence in the 6.5%. In addition, patients who were treated via a curettage of the lesions presented more frequently with deformities after healing, new fractures after surgery, residual pain, non-integration of the graft, and partial healing. Limb length discrepancy was a common complication observed in both treatment groups. In a retrospective analysis conducted by Flont et al. with 26 patients, including 16 undergoing curettage and 10 undergoing en bloc resection, no significant difference was observed between the two groups regarding postoperative symptoms. However, pain, limited range of motion, limb length discrepancy, and a reduction in muscle strength were more prevalent in patients undergoing resection [64], which differs from the findings of our study. This discrepancy could also be attributed to a considerable disparity in the number of patients treated with curettage compared to those undergoing resection, 124 vs. 15. In addition, en bloc excision is a more technically demanding procedure and its success may depend on the skill of the surgeon. In a comparative study by Gibbs et al., no recurrences were reported in patients undergoing en bloc excision compared to a 12% recurrence rate in those treated with curettage [65]. Consistent with the literature, the results of our study allow us to assert that, although both curettage and en bloc excision have their merits and drawbacks, the lower recurrence rate associated with en bloc excision makes it a viable option, especially for recurrent lesions or cases with extensive bone involvement, particularly in expendable areas [64]. Careful consideration of patient-specific factors and surgical expertise is crucial in determining the most appropriate treatment approach.

As previously discussed, only eight cases presented recurrence. This data, which might initially provide optimism regarding the various treatments for pathological fractures in ABC, may be influenced by the short follow-up period considered in several studies. In fact, 18 studies have a follow-up period equal to or less than two years. In the literature, it has been observed that the highest recurrence rate occurs after the initial 2 years [59,60,63]. Hauschild et al., in a review involving a total of 790 patients with ABC with and without pathological fracture, reported a recurrence rate ranging from 12% to 59%; more specifically, in 90% of cases, recurrence occurred within the first year, rarely extending beyond 4 years postsurgery [59].

In all the studies considered, the patients who experienced a recurrence of the ABC were pediatric and less than 18 years old. The literature shows that many aneurysmal bone cysts occur near open physes or articular cartilage in skeletally immature patients [65,66]. This particular location near these crucial growth structures increases the risk of recurrence, largely due to the cautious approach taken in treatment to avoid damaging these critical areas [28,62]. In the literature, we can find that the most common complication when physes are not preserved is physeal arrest with limb length discrepancy and deformity, which can be a challenge for the surgeon to manage. Lin et al. [67] retrospectively studied 53 patients with aneurysmal bone cysts treated between 1989 and 2004 and 10 patients (18.9%) had local recurrence, all with a juxtaphyseal and periarticular localization. They did find a significant association between age and recurrence, with patients aged 12 years or younger being more likely to experience recurrence [67]. In essence, this data suggests that the highest risk of recurrence in ABCs is observed in pediatric patients with lesions located near growth plates or within the proximity of joints. As we already stated, this is often compounded by the tendency to opt for less aggressive surgical approaches in these cases, primarily to mitigate the risk of damaging the growth plates or articular cartilage. Therefore, while treatment decisions must always consider the individual patient and their specific circumstances, these findings underscore the importance of carefully balancing the need for effective treatment with the preservation of critical anatomical structures in pediatric cases of ABCs.

After curettage, the resultant defect is commonly filled using a bone graft or less frequently (only four patients in our series) bone cement, like polymethylmethacrylate (PMMA). Studies reported similar rates of recurrence when using a bone graft or cement, so the literature does not highlight either option [7].

Autograft, allograft, synthetic bone, or a combination were used, as we explain in the results. Among patients from our series for whom the type of graft is specified, an autologous bone graft from the fibula or iliac crest was the most used; an autologous graft was harvested from the tibia in just one patient. On the other hand, for 24 patients, a synthetic bone substitute was chosen, but these data are limited by the fact that, in many articles considered, the authors did not specify the type of graft used, so we can extract only partial conclusions.

Although, in our series, autografts are the most used, they present some limitations that may have induced other authors to use synthetic bone grafts: sometimes they are insufficient to fill large defects and the harvesting process can lead to morbidity in the donor site [41].

Previously, various bone substitutes used as grafts were demonstrated to be effective to fill bone defects after curettage, so they represent a valid alternative to limit the complications described [68].

Ozaki et al. reported less recurrence after cementation compared with bone grafting after curettage [69]; also, in our analysis, none of the patients treated with cement to fill the bone loss after curettage experienced recurrence. However, the thermal effect of the bone cement can determine an injury of the growth plate and the subchondral bone [68], preventing authors from using it in a pediatric population of patients with open physis.

In all patients with lesions located in the femur, except for one which was treated with selective transcatheter arterial embolization both at the neck and trochanteric region, the treatment involved synthesis. Indeed, the presence of a pathological fracture at the level of the proximal femur requires treatment with a fixation device to avoid potential complications such as varus deformity [41,70]. The fixation devices chosen are intramedullary nails, dynamic hip screws (DHSs), or a plate and screws and external fixator. Of these, only one patient developed complications; specifically, the patient treated with an external fixator developed limb length discrepancy. Kushwaha et al. opted for this treatment due to the extent of the lesion reaching the physis area; thus, involvement of the growth plate could justify the complication [27]. Therefore, the use of intramedullary nails in growing patients is debated due to the involvement of the growth plate. Weber et al., in trochanteric fractures, suggest considering not only the lesion’s location but also its size and bone stock. Large lesions and inadequate bone stock should be treated using modular tumor prostheses, while good bone stock allows for the use of intramedullary nails [29]. In none of our studies was treatment with prostheses considered, likely due to the average age of 19.7 years and, thus, the tendency to opt for a less aggressive approach. In our series, 23 studies focusing on the treatment of pathological fractures in the lower limb ABCs, a common thread emerges: a subsequent period of immobilization, typically spanning approximately 11 weeks, is recommended. Indeed, for Beris et al. and Rahman et al., immobilization serves a crucial purpose; it provides the necessary time for the injury to heal effectively, while also serving as a proactive measure to prevent any potential recurrence or exacerbation of fractures [36,50].

In the literature, there is a lack of evidence regarding the optimal method of synthesis to use when dealing with a pathological fracture in a lesion of the lower limbs. Certainly, the choice of surgical approach will primarily depend on the location of the lesion. For instance, in the trochanteric region, a nail might be preferred; meanwhile, in the femoral neck region, options such as a dynamic hip screw (DHS) or screws could be considered, As the results of the analysis of our series of patients show, internal fixation with a plate and screws is the most common form of fixation used, considering that the femoral neck region is the most involved localization. The age of the patient is also a factor to consider, along with the capabilities of the surgeon.

Among the seven patients with lesions located in the spine, 43% of them developed a complication. Specifically, they developed anterolisthesis, worsening of the Cobb angle, and neurological deficits. Geffroy et al. define embolization as the most appropriate treatment for lesions in the lumbar vertebrae, unless there are preoperative signs of neurological involvement or bone involvement leading to deformity [43]. In their study, the two patients with pathological fractures were treated with total vertebrectomy followed by autologous grafting with cortical and cancellous bone to ensure normal vertebral height and osteosynthesis. Only one patient developed a postoperative deformity, but no neurological symptoms were reported. Among the cervical-level injuries, only one case presented complications, specifically marked as kyphosis. The surgical treatment chosen in the study of Welk et al. involved curettage, laminectomy, and the application of a rigid collar; only after developing the complication, the patient underwent osteosynthesis [42]. Kyphosis arises due to the removal of the posterior elements, which contribute significantly to bearing of the cervical weight. Moreover, when there is more extensive removal of the lamina and disruption to the apophyseal joint capsule, the risk of acquiring kyphosis increases. This risk is particularly heightened in pediatric patients due to their cervical ligamentous structures exhibiting greater laxity compared to adults [42]. In a case series conducted by Mehlman et al., it was found that 45% of pediatric patients who underwent a laminectomy developed spinal deformities. Among those patients, 60% required subsequent spinal fusion procedures [71]. In our series, we observed that patients who initially underwent osteosynthesis surgery did not experience any complications. This finding, combined with insights from the broader literature, leads us to the conclusion that relying solely on curettage or resection along with adjunctive measures may not be adequate in vertebral locations. While it is prudent to minimize invasiveness in such delicate areas, it is crucial to recognize the necessity of fusion procedures to mitigate the risks of complications, such as deformities and ensuing neurological symptoms.

Therefore, as can be inferred, both from our results and from the literature, that the higher rate of complications is due to the localization and consequent damage that various surgical techniques can cause. For this reason, over the years, efforts have been made to introduce so-called minimally invasive techniques such as embolization, argon beam coagulation, high-speed burr, and the use of adjuvants such as phenol, polymethylmethacrylate, and liquid nitrogen. These treatments stem from the hypothesis that the development of ABCs has a vascular origin. Often, these treatments are used in conjunction with curettage and/or resections or are used in an initial surgical stage to reduce the size of the lesion, making subsequent treatment less aggressive.

More rarely, these nonsurgical treatments are used alone to manage a pathological fracture in patients with ABC: this is the case in the study by Rossi et al., the only one to use arterial embolization as the sole treatment [46]. However, the patient developed a limb length discrepancy of about 3 cm. In excessively large ABCs, preoperative arterial embolization should be conducted one day before surgery to minimize blood loss during the operative procedure and to facilitate successful curettage [7,28].

Currently, adjuvant agents offer a promising avenue for percutaneous intralesional injection, presenting a significant therapeutic option. Combining adjuvants with curettage seems to enhance the local control rate [9]. Among the widely utilized adjuvants for both intralesional curettage and percutaneous injection are sclerosant agents like Polidocanol and Ethibloc [17,19]. Rastogi et al. demonstrated an impressive 97% cure rate with percutaneous injections of Polidocanol [17]. Sclerotherapy has emerged as a safe method, boasting good local control with minimal side effects [9,19]. Additionally, Varshney et al., in a retrospective study, assumed that patients opting for sclerotherapy often necessitated a higher frequency of repeat procedures to achieve complete healing, contrasting with those undergoing curettage [72]. Ethibloc is a local fibrogenic and thrombogenic agent that serves as a potential sclerotherapy agent, but it has been linked to severe complications, including pulmonary embolism and aseptic [62]. Adamsbaum et al. conducted a review of 17 ABC patients treated with Ethibloc injection and after a 5-year follow-up; complete healing was achieved in 14 patients. However, sixteen patients experienced local inflammatory reactions, and three developed small cutaneous fistulae [73]. Doxycycline also stands out as a potential adjuvant due to its anti-neoplastic properties [19].

An interesting finding within our series was that only two patients subjected to combined treatment experienced a recurrence of the lesion. This insight underscores the significant role that the aforementioned treatments may play in influencing the likelihood of lesion recurrence.

Regarding postoperative follow-up, it was not possible to analyze and compare a clinical or functional score in order to establish any conclusion about the most effective treatment for the patients in terms of outcome. Out of the thirty-seven articles examined, only the results of eight patients were evaluated through a score and only four articles reported the same score through which to make a reliable comparison possible. Furthermore, most of the clinical/functional scores were specific for the outcome assessments of a particular localization, making a comparison between all patients difficult due to the variety of locations considered.

Defining a gold standard surgical approach for pathological fractures in ABCs remains highly challenging today. One significant factor to consider is the lesion’s location, distinguishing between weight-bearing and non-weight-bearing areas, which can indicate the necessity of fixation devices. Patient age can also influence the choice, particularly if the lesion affects juxtaphyseal and periarticular regions, potentially leading to complications such as limb length discrepancies and varus–valgus deformities in pediatric patients with open physes.

Even among adjuvant therapy, it is difficult to choose the best option because there are no comparative studies regarding adjuvant agent efficacy and the literature consists largely of case series from single institutions.

Analyzing the data presented, we can state that a multidisciplinary approach is the best solution for the management of patients with pathological fractures in ABC, as has already been proposed in the literature [74]. In the majority of cases, this multidisciplinary management is based mainly on surgery followed by adjuvant therapy administered by the oncologist or embolization performed by the intervening radiologist. Finally, even pharmacological treatments can help to control associated symptoms in our patients. Therefore, it is crucial to present each case to a multidisciplinary board to plan the best procedures for optimizing our patients’ outcomes.

This review presents some limitations due to the fact that most of the articles taken into account have a low Coleman score and are case reports of single patients’ experiences. Other important limitations in our study include the absence of risk of bias assessment for the included studies and the lack of a meta-analysis due to data heterogeneity. Most of the studies taken into account considered a small number of patients, with a short follow-up, treated based mainly on the experience of the surgeon rather than on objective diagnostic–therapeutic algorithms; therefore, all the factors associated with the absence of a endorsed guideline can lead to bias. This consideration impacts the possibility to suggest a gold-standard treatment. On the other hand, this review gathers different surgical approaches that can be adopted according to the localization of the cyst and the age of the patient, tailoring the treatment in order to maximize the healing rate. It would be helpful for future research to conduct more retrospective comparative studies in order to provide a better comparison between different techniques and, thus, effectively evaluate which treatment might be the best.

## 5. Conclusions

In conclusion, the treatment of pathological fractures in aneurysmal bone cysts requires careful patient assessment, considering factors such as age, the presence of open growth plates, the location of the lesion, and the surgeon’s expertise. Curettage has traditionally played a crucial role in cyst treatment, and additional interventions such as embolization, high-speed burring, and adjuvant can be adjuncts to reduce recurrence rates, which have proven to be the most frequent complication. Additionally, the use of fixation devices in lower limb fractures is essential.

Due to the low quality of the studies in the literature (most of the studies analyzed have a low Coleman score), we are unable to define a univocal and reliable therapeutic algorithm to follow.

## Figures and Tables

**Table 2:** Patient treated with curettage.

Authors	Patients Treated	Reconstruction	Adjuvants	Type of Synthesis
Sonkusale et al. [24]	1	Hydroxyapatite	-	Plate
Pai et al. [25]	1	Bone graft (N/S)	-	Plate
Teixeira-Vaz et al. [26]	1	Bone graft (N/S)	Cryotherapy	Nail
Kushwaha et al. [27]	1	Autologous bone graft	-	External fixator
Tomaszewski et al. [28]	6	Bone graft (allogenic + synthetic)	-	Plate
Weber et al. [29]	1	Bone graft (N/S)	-	Nail
Trăilescu et al. [30]	3	Hydroxyapatite tricalcium phosphate	-	TEN
Dorosh et al. [31]	1	Calcium sulphate calcium phosphate	-	Nail
Okuda et al. [33]	1	Bone graft (autologous + synthetic)	Calcitonin Prednisolone	TLIF
Kirker et al. [34]	1	Bone graft (autologous + calcium phosphate	-	ORIF
Rahman et al. [36]	4	Bone graft (autologous)	Liquid nitrogen injections	Internal fixation
Panchwagh et al. [37]	4	Bone graft (autologous)	-	Plate Cannulated screw
Arif et al. [38]	1	Bone graft (autologous)	-	-
Ferreira et al. [39]	1	-	Electrocauterization	-
Plaikner et al. [40]	1	Bone graft (autologous)	-	-
Erol et al. [41]	59	Allogenic bone graft (*n* = 41) Autologous bone graft (*n* = 4) Allogenic bone + autologous bone graft (*n* = 12) Cement (*n* = 2)	-	(N/S, *n* = 34)
Welk et al. [42]	1	-	-	-
Babazadeh et al. [44]	1	Autologous bone graft + synthetic bone graft	Phenols Alcohols	Plate
Rapp et al. [45]	2	Synthetic bone graft	Autologous platelet-rich plasma	Nail
Xu et al. [48]	1	Autologous bone graft	-	Screws
Nydick et al. [49]	1	Synthetic bone graft+ Demineralized bone matrix	-	-
Beris et al. [50]	1	Autologous bone graft	-	Plate
Clayer et al. [51]	15	Calcium sulphate	-	-
Lampasi et al. [52]	1	-	-	-
Goddard et al. [53]	1	-	-	Bars
Jackson et al. [22]	3	Bone graft (N/S)	-	-
Ortiz et al. [54]	7	Bone graft (N/S)	-	-
Session et al. [55]	1	Cement	Phenol	-
Gailloud et al. [56]	1	PMMA Barium powder	-	-
Yamamoto et al. [58]	1	Hydroxyapatite	-	-

**Table 3:** Patient treated with en bloc resection.

Authors	Patients Treated	Reconstruction	Adjuvants	Type of Synthesis
Purohit et al. [35]	1	Autologous bone graft	-	Plate
Chhawra et al. [32]	1	-	Phenols	-
Grzegorzewski et al. [47]	2	Autologous bone graft	-	-
Schmitz et al. [23]	1	Autologous bone graft	-	Arthrodesis
Snell et al. [57]	1	Autologous bone graft	-	Arthrodesis
Geffroy et al. [43]	2	Autologous bone graft	-	Arthrodesis
Erol et al. [41]	5	Endoprosthetic or biological (*n* = 2)	-	(N/S, *n* = 34)
Ortiz et al. [54]	2	Autologous bone graft (*n* = 1)	-	-

**Table 4:** Complications.

Authors	Patients Treated	Recurrence	Bone Shortening	Angular Deformities	Fractures	Others
Pai et al. [25]	1	1 (100%)	-	-	-	-
Tomaszewski et al. [28]	6	1 (16.67%)	-	-	-	-
Purohit et al. [35]	1	-	1 (100%)	-	-	-
Rossi et al. [46]	1	-	1 (100%)	-	-	-
Xu et al. [48]	1	-	1 (100%)	-	-	-
Babazadeh et al. [44]	1	-	-	-	-	1 (100%)
Chhawra et al. [32]	1	-	-	-	-	1 (100%)
Grzegorzewski et al. [47]	1	-	1 (100%)	-	-	-
Welk et al. [42]	1	-	-	1 (100%)	-	-
Geffroy et al. [43]	2	-	-	1 (50%)	-	1 (50%)
Erol et al. [41]	64	2 (3.12%)		3 (4.7%)		2 (3.12%)
Clayer et al. [51]	15	2 (13.3%)	-	-	3 (0.2%)	1 (6.67%)
Ortiz et al. [54]	9	2 (22.2%)	-	-	-	2 (22.2%)
Kushwaha et al. [27]	1	-	1 (100%)	-	-	-

## Data Availability

The datasets used and/or analyzed during the current study are available from the corresponding author on reasonable request.
